# Dependence of treatment planning accuracy in peptide receptor radionuclide therapy on the sampling schedule

**DOI:** 10.1186/s13550-016-0185-8

**Published:** 2016-03-25

**Authors:** Christian Maaß, Jan Philipp Sachs, Deni Hardiansyah, Felix M. Mottaghy, Peter Kletting, Gerhard Glatting

**Affiliations:** Medical Radiation Physics/Radiation Protection, Universitätsmedizin Mannheim, Medical Faculty Mannheim, Heidelberg University, Theodor-Kutzer-Ufer 1-3, 68167 Mannheim, Germany; Department of Nuclear Medicine, Ulm University, Ulm, Germany; Klinik für Nuklearmedizin, University Hospital, RWTH Aachen University, Aachen, Germany; Department of Nuclear Medicine, Maastricht University Medical Center (MUMC+), Maastricht, The Netherlands

**Keywords:** Physiologically based pharmacokinetic (PBPK) modeling, Peptide receptor radionuclide therapy (PRRT), Optimal sampling schedule, Neuroendocrine tumor (NET), Time-integrated activity coefficient (TIAC)

## Abstract

**Background:**

Peptide receptor radionuclide therapy (PRRT) plays an important role in the treatment of neuroendocrine tumors (NET). Pre-therapeutic dosimetry using the area under the measured time-activity curve (AUC) is important. The sampling schedule for this dosimetry determines the accuracy and reliability of the obtained AUC.

The aim of this study was to investigate the effect of reduced number of measurement points (i.e., gamma camera image acquisition or serum measurements) on treatment planning accuracy in PRRT using ^111^In-labeled-diethylenetriaminopentaacetic acid-octreotide (DTPAOC; Octreoscan™).

**Methods:**

Pre-therapeutic biokinetic data of 15 NET patients were investigated using a recently developed physiologically based pharmacokinetic (PBPK) model. Two parameter sets were determined (standard or iterative approach) and used for calculation of time-integrated activity coefficients (TIACs) for the tumor, kidneys, liver, spleen, serum, and whole body. TIACs obtained using the full data sets were used as reference. To evaluate the effect of sampling on individual treatment planning, reduced sampling schedules were generated omitting either 1, 2, 3, or 4 organ and serum measurements or all serum measurements for each patient. Relative deviations (RDs) between these and reference TIACs were calculated and used as criterion for treatment planning accuracy. An RD < 0.1 was considered acceptable.

**Results:**

When omitting serum measurements, TIAC accuracy remained acceptable (RD < 0.1) for the standard approach. The kidney TIACs could be estimated for both approaches with acceptable RDs using two time points (*t* = 4 h; 2 d); tumor RDs were <0.3. The iterative approach reduced the range of RD, but did not further reduce the number of needed measurement points (i.e., to achieve an RD <0.1). For both approaches RDs for liver, spleen and whole body were larger than 0.1. However, in the clinical setting these RDs are less relevant as liver and spleen are not organs at risk due to the low absorbed doses.

**Conclusions:**

When using a priori information of a PBPK model structure combined with Bayesian information about PBPK model parameter distribution, the administered activity could be determined with acceptable accuracy using only two time points (4 h, 2 d) and thus allow a considerable reduction of needed data for individual dosimetry.

## Background

Peptide receptor radionuclide therapy (PRRT) is performed in the treatment of neuroendocrine tumors (NET), where somatostatin analogues (DOTATOC, DOTATATE) are radiolabeled with ^177^Lu, ^90^Y, or ^111^In for pre-therapeutic and therapeutic purposes [1–3]. Treatment planning is based on pre-therapeutic measurements using ^111^In-labeled analogues, especially when ^90^Y is used as a radiolabel for therapy [4]. Several nuclear medicine centers determine the therapeutic absorbed dose based on the area under the pre-therapeutic time-activity curve [5–7]. These areas are normalized by the administered activity yielding time-integrated activity coefficients (TIACs; formerly: residence times [4]), which are directly proportional to the absorbed doses. Thus, for a precise evaluation of the therapeutic absorbed doses, a reliable and accurate estimation of these areas is essential. This is performed by applying a model, e.g., a sum of exponential functions or physiologically based pharmacokinetic (PBPK) models [8]. Recent work [9] showed pre-therapeutic and therapeutic biodistributions may change depending on the injected amount of peptide as well as the used peptide. In using a PBPK model, these changes can be described and predicted. Additionally, a PBPK model allows implementation of a priori information, which increases the ratio of number of measurements and fit parameters. A value for that ratio of >3 [8] is sufficient for accurate determination of parameter values and AUCs. On the contrary, using, e.g., five measurement points, this value is 2.5 or 1.25 if using 1 or 2 exponentials.

The model parameters influence the estimation of the area under the time-activity curves (AUCs). A reliable and accurate estimation of the fit parameters in turn is affected by the sampling schedule [10–13].

The reduction of the number of required measurement time points would reduce patient burden and radiation exposure to staff. Therefore, it is important to know the dependence of the sampling schedule on treatment planning accuracy.

One aspect of improving the prediction accuracy of a PBPK model is using a priori knowledge, for example, the information about the parameter distribution in the investigated patient population. This information can be introduced into a PBPK model as Bayes parameter values, i.e., an implementation of population mean values and their respective standard deviation. In treatment planning of leukemia patients using radiolabeled anti-CD66 antibodies, this improved model performance and allowed an accurate prediction of biodistribution of the therapeutic agent [14].

However, population information, e.g., in the form of Bayes parameters [15–18], has not yet been implemented into a PBPK model to optimize or simplify the sampling schedule, i.e., to reduce the number of measurements without scrutinizing estimation accuracy.

The aim of this work was to investigate the effect of sampling schedules with fewer measurement points on treatment planning accuracy for PRRT using ^111^In-labeled diethylenetriaminopentaacetic acid-octreotide (DTPAOC; Octreoscan™, Mallinckrodt plc, Chesterfield, UK) using a PBPK model and Bayes parameters. Two different algorithms were employed to determine the Bayes parameter values.

## Methods

### Data acquisition

In total, data of 15 patients with proven neuroendocrine tumors (NETs) were included in the study. Radiolabeling was performed as described elsewhere [9]. Planar whole-body scans using a double-head γ-camera (Siemens, Erlangen, Germany) were performed at 0.75, 4 h, 1, 2, and 3 or 5 d after the administration of 8.1 ± 0.5 μg radiolabeled DTPAOC (Octreoscan™, Mallinckrodt plc, Chesterfield, UK) with a mean ^111^In activity of 143 ± 17 MBq for prediction of the absorbed doses [9]. The quality control of the product was performed as described in the instruction leaflet. In all cases, the radiochemical purity was above 98 %. Images were attenuation and scatter corrected as described in [9]. Organ activity values were determined for accumulating organs using ULMDOS software [19], with scatter correction and an effective attenuation correction based on the first whole-body measurement in conjugate view mode. Blood samples were drawn at 5 min, 0.5, 1, 2, 4 h, and 1, 2, 3, or 5 d.

### PBPK model

To describe the biodistribution of radiolabeled peptides, a recently developed PBPK model [9] was used. Physiological processes, e.g., absorption, distribution, internalization, and excretion (ADME) were implemented. Distribution of the injected peptide was modeled via blood flow circulating between the organs (tumor, kidneys, liver, and spleen). These organs show high somatostatin receptor subtype 2 (sst2) expression and/or unspecific uptake (mainly the kidneys). Free passage of the peptide between interstitial and vascular spaces was assumed for the tumor, liver, and spleen [20]. Other organs were merged into one compartment (rest).

The intravenous injection of the radiolabeled compound into the patients was modeled as a bolus injection in the main blood compartment.

The PBPK model consists of two equal subsystems, which are connected by the competition of labeled and unlabeled peptide for the same receptors and physical decay of the label. Specific binding to somatostatin receptors subtype 2 and degradation of bound peptide was considered. The kidney model describes unspecific uptake as well as release of still intact peptide back into the serum [9].

### Parameter fitting

Within the PBPK model, different types of parameters were used: Model parameters refer to all parameters used in the PBPK model, may they be fitted or fixed parameters. Fitting parameters are those of the model parameters whose values are estimated in the fitting process. They can be set as adjustable or Bayes parameters. Adjustable parameters may have an upper and lower bound but no further constraints, whereas Bayes parameters have a constraint in the form of a mean value and a standard deviation.

To evaluate the impact of a priori population information on optimizing the sampling schedule (i.e., to reduce the number of measurement points), two different algorithms were employed to determine population Bayes parameter values. For the first algorithm (standard approach), the software Simulation, Analysis, and Modeling (SAAM II; version 2.3, The Epsilon Group, Charlottesville, USA) was used [21]. This software provides a framework for model development. Using the built-in Rosenbrock optimization algorithm, the model parameters were fitted to biokinetic data.

For the second algorithm (iterative approach), the software PopKinetics (version 2.2, The Epsilon Group, Charlottesville, USA) was used. For both approaches, the same optimizer was used and the main difference is how Bayes parameters were determined. A more detailed description of both algorithms is presented in the following subsections.

All organ activity measurements were used during the fitting process (simultaneous fitting of all data). However, the effects on TIAC estimation accuracy were only evaluated for the tumor and kidney as these are the target and dose-limiting organs of this therapy.

Either set of Bayes parameter values derived from the standard and iterative approach algorithms was implemented as starting values in the PBPK model to predict time-integrated activity coefficients for different sampling schedules.

#### Standard approach

Model parameters were fitted to pre-therapeutic biokinetic data (gamma camera and blood samples) of each patient individually (Table 1). In this approach, a combination of individual (adjustable) and population (Bayes) parameters were used: the receptor numbers for the tumor *R*_T_, kidneys *R*_K_, liver *R*_L_, remainder of body *R*_R_, the tumor volume *V*_T_, the degradation rate of bound peptide in the tumor *λ*_deg, T_, and the glomerular filtration rate GFR were implemented as adjustable parameters; the degradation rates for kidneys *λ*_deg, K_, liver *λ*_deg, L_, spleen *λ*_deg, S_, remainder of the body *λ*_deg, R_, the relative blood flow to the tumor *f*_T_, and the relative excretion fraction by the kidneys *f*_ex_ were implemented as Bayes parameters. The starting values for these parameters were implemented as population mean and standard deviation as reported in recent work [9]. Computational settings were selected as described elsewhere [9].

**Table 1:** Model parameters and start values of the fitting parameters

Parameter	Standard approach	Iterative approach
*R*_K_ [nmol]	1.4^a^	2.1 ± 0.8^b^
*R*_L_ [nmol]	2.1^a^	1.8 ± 0.7^b^
*R*_S_ [nmol]	3.8^a^	3.5 ± 3.1^b^
*R*_R_ [nmol]	6.4^a^	7.3 ± 2.5^b^
*V*_T_ [l]	0.3^a^	0.2 ± 0.3^b^
*f*_T_ [ml/min/g]	0.2 ± 0.1^b^	0.25 ± 0.06^b^
*f*_ex_ [%]	96 ± 1.0^b^	96^c^
GFR [ml/min]	57.4^a^	64.4 ± 15.1^b^
*λ*_deg,T_ [10^−4^/min]	1.1^a^	1.5 ± 0.6^b^
*λ*_deg,K_ [10^−4^/min]	1.1 ± 0.1^b^	1.1^c^
*λ*_deg,L_ [10^−4^/min]	0.5 ± 0.1^b^	0.5^c^
*λ*_deg,S_ [10^−4^/min]	1.6 ± 0.1^b^	1.6^c^
*λ*_deg,R_ [10^−4^/min]	1.3 ± 0.1^b^	1.3^c^
# of fitting parameters	13	8

^a^Adjustable parameters

^b^Bayes parameters with mean ± SD

^c^Fixed parameter values

#### Iterative approach

First, model parameters were fitted to biokinetic data of each patient individually as described in the standard approach above. Second, population mean and standard deviation for all parameters were calculated. Subsequently, these values were introduced as Bayes parameter values for each individual patient, constraining the possible parameter values based on the mean and standard deviation. Iteratively, the algorithm fitted the model parameters anew and updated the values until the convergence criterion or the maximum number of iterations was reached [14]. In contrast to the standard approach, this iterative approach includes only Bayes parameters.

The convergence criterion of the implemented algorithm was set to 10^−4^, relative and absolute error to 0.01 and 0.1, respectively. The maximum number of iterations was set to 200, and a data-based variance model was chosen. These estimated population parameters were then used as starting values (Table 1).

### Sampling schedule

**Fig. 1:**
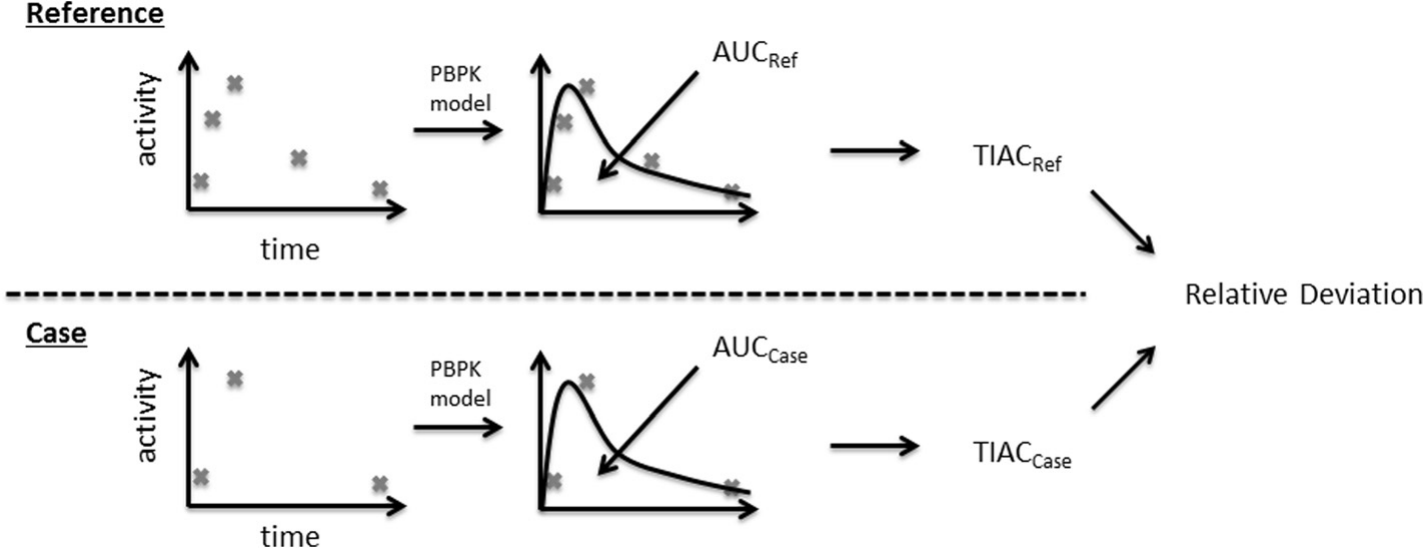
Schematic overview of the workflow. Based on existing activity measurements, a reference and reduced sampling schedule (“case”) were generated. The reference sampling schedule includes all available biokinetic data for a given patient. To describe the biokinetics, the parameters of the employed PBPK model were fitted. After parameter fitting (standard or iterative approach), the areas under the time-activity curves were determined and subsequently, the time-integrated activity coefficients were calculated. The same methodology is applied to the sampling schedules omitting alternated numbers of measurement points. At the end, the derived time-integrated activity coefficients (TIACs) are quantitatively compared and evaluated for treatment planning accuracy by calculating the relative deviation (Eq. (1))

To investigate the effect of the altered sampling schedules on the estimation accuracy of TIACs in PRRT, biokinetic data of each patient were modified in five cases (Table 2; Fig. 1): omitting either one up to four time points for every organ and the corresponding serum measurements (case I–IV) or omitting all serum measurements but including all organ time points (case V). As there are eight serum measurements but only five organ measurements, serum measurements were omitted with respect to the corresponding (time-wise) organ measurement(s). For example, omitting an organ measurement at *t* = 2 d, the corresponding serum measurement at the same time was omitted. However, for the first organ measurement (*t* = 0.75 h), the serum measurement at *t* = 1 h was omitted. Other possible combinations of omitting organ and serum measurements were not investigated.

**Table 2:** Overview of the investigated sampling schedules

Case	Measurement	Number of omitted time points	Number of combinations^a^	Total no. of measurements^b^	Ratio of no. of measurements to fit parameters	
					Standard approach	Iterative approach
Reference	Organ + serum	0	1	33	2.5	4.1
I	Organ + serum	1	5	27	2.1	3.4
II	Organ + serum	2	10	21	1.6	2.6
III	Organ +serum	3	10	15	1.2	1.9
IV	Organ + serum	4	5	9	0.7	1.1
V	Organ	8 (serum)	1	25	1.9	3.1

^a^The number of different sampling schedules when omitting each measurement point once

^b^Five organ data per time point and 8 for serum measurements

These artificially generated reduced sampling schedules (total 31/patient) were then implemented in the PBPK model, and the model parameters were fitted again to these data (using either standard or iterative Bayes parameter values as a priori knowledge, Table 1). For each case, AUCs for the tumor, kidneys, liver, spleen, serum, and whole body were calculated by integration of the fitted curves (over 14 d). The obtained AUC values were divided by the amount of radiolabeled substance administered to determine TIACs individually.

### Validation and statistical analysis

Reference TIACs *ã*_ref_ were determined patient-wise by using the full data set (providing maximum information) and the parameter values determined by the standard approach. TIACs based on the altered sampling schedules *ã*_fit_ were calculated for both approaches. Treatment planning accuracy was quantitatively evaluated by calculating the relative deviation (RD) according to:

1 $$ \mathrm{R}\mathrm{D}=\left({\overset{\sim }{a}}_{\mathrm{fit}}{\textstyle \hbox{-} }{\overset{\sim }{a}}_{\mathrm{ref}}\right)/{\overset{\sim }{a}}_{\mathrm{ref}}. $$

An RD < 0.1 was considered as an acceptable deviation in treatment planning accuracy in any further analysis.

Coefficients of variation (CV; ratio of standard deviation over the corresponding value) were calculated for all investigated cases to investigate the accuracy of the estimated TIACs.

An overview of the workflow is presented in Fig. 1.

Additionally, the adjusted *R*^2^ values were calculated for all cases and both approaches (i.e., standard and iterative).

## Results

Visual inspection showed good fits for all cases and both parameter sets. Accordingly, the adjusted *R*^2^ value was larger than 0.9. The population means and standard deviations for the individual time-integrated activity coefficients *ã*_ref_ using the standard parameter values were 2.5 ± 4.3, 1.2 ± 0.3, 1.5 ± 0.5, 1.9 ± 1.1, 14.6 ± 4.1, and 0.9 ± 0.1 h for the tumor, kidneys, liver, spleen, whole body, and serum, respectively.

CVs of the estimated TIACs were calculated for all cases and were precise (<25 %) or acceptable (<50 %), except for cases III and IV (Table 2) using the standard parameter set.

Relative deviations (RD) were calculated for all cases and averaged over all organs and all patients. The lowest RD (best case) and largest RD (worst case) for every investigated case are presented in Table 3. The corresponding CVs for the lowest RD are presented in Table 4.

**Table 3:** Mean relative deviations of the fitted and reference TIACs averaged over all patients and all organs. For cases I to IV, the combination of omitted measurement points that gave the best and worst results in terms of the lowest and largest relative deviation (above and below the dashed line) are presented

RD [%]	Standard approach				Iterative approach			
Case	Omitted time point	Mean ± SD	Min	Max	Omitted time point	Mean ± SD	Min	Max
0	–	(Reference)			–	1 ± 5	−6	13
I	2nd	0 ± 4	−6	9	4th	2 ± 6	−10	13
	4th	−1 ± 6	−16	9	3rd	0 ± 11	−19	26
II	2nd, 4th	0 ± 6	−11	13	1st, 3rd	0 ± 7	−14	15
	3rd, 5th	6 ± 39	−36	133	3rd, 5th	1 ± 11	−13	31
III	1st, 3rd, 5th	5 ± 17	−13	51	1st, 3rd, 5th	0 ± 8	−12	18
	2nd, 3rd, 5th	22 ± 58	−26	175	3rd, 4th, 5th	22 ± 86	−34	309
IV	–^a^				1st, 2nd, 4th, 5th	6 ± 13	−15	37
	–^a^				1st, 3rd, 4th, 5th	16 ± 55	−26	188
V	1st–8th^b^	0 ± 3	−7	9	1st–8th^b^	1 ± 5	−6	14

^a^No fitting was performed for the standard approach, because an equal number of data and fitting parameters were present

^b^Only serum measurements omitted

**Table 4:** Coefficients of variation (CV) of the estimated TIACs for the best cases (Table 3)

CV [%]	Standard approach					Iterative approach		
Case	Omitted time point	Mean ± SD	Min	Max	Omitted time point	Mean ± SD	Min	Max
I	2nd	7 ± 5	1	22	4th	7 ± 4	1	18
II	2nd, 4th	9 ± 8	1	28	1st, 3rd	5 ± 4	1	16
III	1st, 3rd, 5th	8 ± 9	1	31	1st, 3rd, 5th	6 ± 6	1	21
IV	–^a^	–^a^	–^a^	–^a^	1st, 2nd, 4th, 5th	6 ± 3	4	16
V	1st–8th^b^	7 ± 6	0	23	1st–8th^b^	7 ± 5	1	20

^a^No fitting was performed for the standard approach, because equal numbers of data and fitting parameters were present

^b^Only serum measurements omitted

The lowest RDs for the tumor and kidneys (averaged over all patients) are presented in Fig. 3. As expected, TIAC accuracy reduces considerably (i.e., larger RDs) when fewer measurement points were taken into account in combination with parameter values derived using the standard approach. However, the application of population-based parameters (iterative approach) helped to reduce the systematic offset and the range of the RD (min, max), especially for the tumor up to three-fold (Table 3). For both approaches, the RDs of the TIACs in the kidneys were similar for all cases.

Overall, the acceptance criterion for the standard approach was fulfilled when omitting the time point *t* = 2 h or when omitting the serum measurements (Table 3). Using the population-based parameter values, the criterion was not fulfilled for any case I–V.

For the kidneys, the criterion was fulfilled in any case when using either standard- or population-based parameter values (averaged over all patients), except for case IV (iterative approach). However, for the tumor, only case V (omitting all serum measurements) for the standard approach fulfilled the acceptance criterion.

The RDs for the liver, spleen, and the whole body did not fulfill the criterion. Moreover, they were not considered in any further analysis, as these organs are neither dose limiting nor express a considerable sst2 receptor density.

## Discussion

In general, the presented PBPK model could describe the investigated data adequately in all cases, i.e., visual inspection and the coefficients of variation for the TIACs were precise or acceptable (Table 4; [22]).

Including population information as a priori knowledge during the fitting process helped to considerably improve prediction accuracy and reduce the corresponding standard deviations when averaged over all organs and all patients (Table 3). When looking at the accuracy of TIAC estimation for tumor (averaged over all patients) for each case (Fig. 3), the application of population information reduced the range of accuracy as well; for the kidneys, the accuracy seems minorly affected indicating that the information about the population implemented in the model was sufficient to compensate for a reduced data set.

**Fig. 2:**
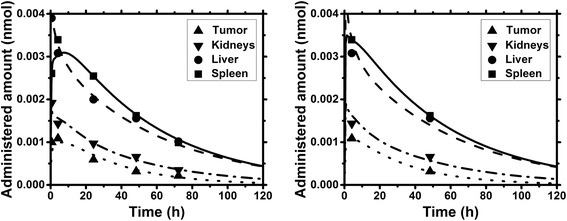
Biodistribution and model fit for a typical patient for the full data set (left) and a reduced data set (right, case III) using the parameter values of the standard approach

The acceptance criterion held true for the kidneys in all cases (except case IV). Typically, the activity to administer is determined based on a maximum absorbed dose to the kidneys. With the presented approach (PBPK model and reduced sampling schedule), an accurate estimation of the absorbed dose in the kidneys may be achieved by using only two measurement time points, i.e., 4 h and 2 d (Fig. 2). This would optimize the clinical workflow considerably (by shortening the sampling schedule) and reduce patient burden and working load of staff. Note that this procedure is only applicable when using a PBPK model as the number of available measurement points (Table 2) is still larger than the number of fitting parameters (13 or 8 for the standard or iterative approach), which would not be the case when using sums of exponential functions for each organ. These results are only applicable for patients undergoing PRRT treatment planning using ^111^In-DTPAOC, and the stated protocol as the biodistribution and, e.g., clearance via the kidneys strongly depends on the radiopharmaceutical [23]. However, using the here presented approach, treatment planning for other radiolabeled compounds may be optimized similarly.

**Fig. 3:**
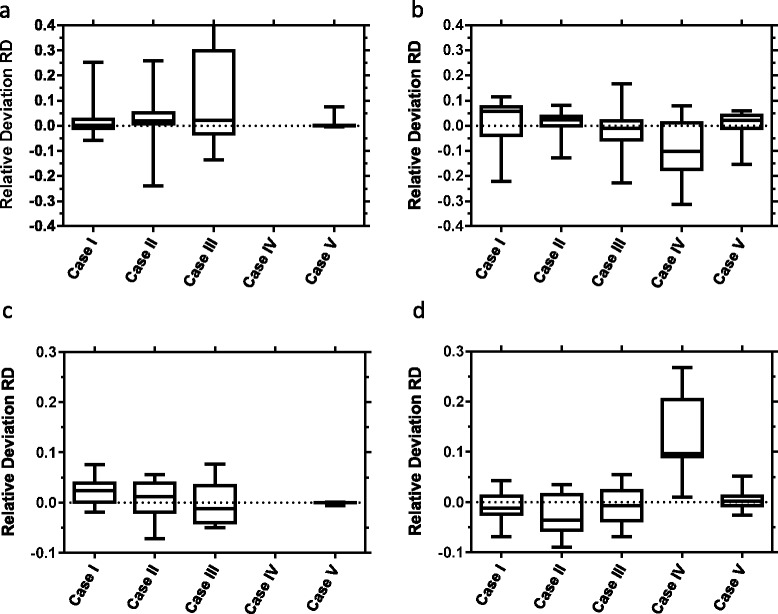
*Box plot* showing median, 25 and 75 % quartiles, min and max of the RD of AUC estimation for the best case (least RD) and the reference data set for the tumor (**a**, **b**) and kidneys (**c**, **d**) indicating mean and standard deviation. *Left panels* show the results for the standard approach (**a**, **c**). Note that for case IV, the number of available data points is smaller than the number of fit parameters. Therefore, a fit could not be performed. *Right panels* (**b**, **d**) show the corresponding results for the iterative approach. Cases I–IV represent omitting 1, 2, 3, or 4 organ and serum measurements; case V represents omitting all eight serum measurements

The acceptance criterion was not fulfilled for the tumor. Therefore, when the calculation of the activity to administer is based on a desired or prescribed dose in the tumor, this approach could over- or underestimate the absorbed dose to the tumor of some patients when omitting already one single time point (Fig. 3). These differences may be due to a large variability in tumor properties, e.g., size, receptor density, and perfusion.

The differences in TIAC estimation for the liver, spleen, and the whole body were not further considered, as these organs are neither dose limiting nor express a considerable sst2 receptor density [24].

Omitting the serum measurements had a minor effect on the accuracy for both the tumor and kidneys for the standard investigation. Therefore, these measurement points may be omitted in future treatment planning, when the same (or at least a sufficiently similar) sampling schedule is considered. Note, however, that these data may nevertheless be needed for proper calculation of red bone marrow absorbed dose.

Based on the provided sampling schedule, the estimated TIACs were set as reference. However, this sampling schedule may be inherently suboptimal. If fewer measurement points are considered, the influence of each single remaining data point will become more prominent. Clearly, the number of fitting parameters limits the number of required measurements to achieve a sufficiently precise estimation of TIACs and hence absorbed doses. When looking at the estimated standard deviations of the fitting parameters (for both approaches, standard, and iterative), these increase with decreasing number of measurements (results not shown). This in turn was reflected in the estimation of the TIACs. Therefore, conducting a precise estimation of TIACs is limited if too many time points (measurements) are omitted. To overcome this problem, simulations could be performed to determine when (in contrast to how often) the biodistribution should be measured. This may reduce the standard deviations of the fitting parameters and may yield a globally optimal sampling schedule.

A possible limitation of the presented study was the number of investigated patients (*N* = 15). To evaluate that the patient population is of sufficient size, e.g., to determine reliable parameter values, the Jackknife resampling method may be applied [14]. This method investigates the impact of a single patient with respect to the full population and to the outcome of the fitting procedure (i.e., parameter values and thus TIACs values).

Further reduction in the size of the measurement data may be achieved by improving the quality of the data, i.e., when using PET imaging or by implementing more a priori knowledge about the population and the investigated disease. Further research could also be aimed at designing more individualized optimal sampling schedules. Subgroups of patients may be based on differences in pharmacokinetic properties (e.g., kidney clearance) or general biodistribution. For these groups, an optimal sampling schedule may be determined by possibly reducing the location and/or number of measurement points even further. However, this approach needs the assumption that patients can be categorized into sufficient different subgroups. This information would also be required a priori to any modeling to ensure that the correct sampling schedule is assigned for a certain patient group.

## Conclusions

The sampling schedule considerably affects estimation accuracy of time-integrated activity coefficients and thus treatment planning for NET patients in PRRT with ^111^In-DTPAOC.

In this patient group, the combination of individual and population information in the PBPK model showed the least effect on the estimation accuracy when the second time point (*t* = 2 h) or all serum measurements were omitted. Thus, in the future treatment planning, these data points may be omitted, if the absorbed dose to the red bone marrow is not of interest. The absorbed dose in the kidneys may be determined with acceptable accuracy using only two time points (4 h and 2 d).

The application of a priori knowledge using the iterated approach and physiologically based pharmacokinetic modeling allowed reduction of the standard deviation of the accuracy, but did not help to further reduce the number of measurement points.

### Ethical approval

All procedures performed in studies involving human participants were in accordance with the ethical standards of the institutional and/or national research committee and with the 1964 Helsinki declaration and its later amendments or comparable ethical standards. Informed consent was obtained from all individual participants included in the study.
